# Small Bowel Adenocarcinoma: A Rare Case of Iron Deficiency Anemia

**DOI:** 10.7759/cureus.32724

**Published:** 2022-12-20

**Authors:** Maira D Nousherwani, Talat Waseem, Umaima M Khattak, Hira Tariq, Mehreen Ashfaq, Maham Babur

**Affiliations:** 1 Surgery, Shalamar Institute of Health Sciences, Lahore, PAK; 2 General Surgery, Shalamar Medical and Dental College, Lahore, PAK; 3 Internal Medicine, Shalamar Medical and Dental College, Lahore, PAK; 4 Community Medicine, Shalamar Institute of Health Sciences, Lahore, PAK; 5 Internal Medicine, CMH Lahore Medical College and Institute of Dentistry, Lahore, PAK; 6 Internal Medicine, Women Medical and Dental College, Abbottabad, PAK

**Keywords:** jejunectomy, segmental resection, iron deficiency anemia, small bowel malignancy, adenocarcinoma

## Abstract

Neoplasms of the small bowel are relatively rare, with less than 5% occurrence among other GI cases. Primary adenocarcinoma, an aggressive sub-type of small bowel cancers, usually presents with vague blood loss and abdominal pain symptoms, causing a delayed diagnosis at an advanced stage of the disease and a poor prognosis. The preferable treatment of choice is surgical resection with chemotherapy, which has shown to have survival benefits. Here we present a case of a 66-year-old male patient with persistent iron deficiency anemia requiring multiple blood transfusions and an unexplained weight loss. A series of diagnostic tests, including upper and lower GI endoscopy, Tc-99 RBC scintigraphy, barium follow-through, CT scans, bone marrow biopsy, esophagogastroduodenoscopy and colonoscopy were inconclusive. He was later diagnosed with a small bowel adenocarcinoma on exploratory laparotomy and surgically treated. Adjuvant chemotherapy was also started. Our case report highlights the course of SBA presenting in an unusual way which was challenging to diagnose with the standard investigations to help physicians/surgeons suspect it at an early stage in the future. This may save patients and help avoid delayed diagnosis or misdiagnosis, especially in patients with an unusual presentation like our patient who presented only with iron deficiency anemia.

## Introduction

Primary adenocarcinoma is a rare chemo-resistant malignant tumor in the small intestine with an aggressive clinical course [1]. It accounts for less than 5% of all gastrointestinal (GI) cancer cases [2]. Of these neoplasms, 55%-82% are present in the duodenum with 11%-25% occurring in the jejunum. The remaining 7%-17% appears in the ileum [3-5]. They are broadly classified as small bowel adenocarcinoma (SBA) and neuroendocrine tumors, each contributing to 40% of the cases with GI stromal tumors. Lymphomas and sarcomas account for 20%-25% [3-5].

It originates from the proliferation of epithelial cells lining the small intestine in the mucosal layer that results in benign polyps that, after a dormant period of 10-20 years, transform into adenocarcinomas [6]. There is a six to eight months' delay in the clinical presentation and diagnosis because an endoscopic inspection of carcinomas is not effective, mainly when they are distal to the duodenum [7,8]. The majority of SBA patients are diagnosed after the onset of symptoms, usually by stenosis-related symptoms such as abdominal pain or vomiting and symptoms related to bleeding. Weight loss, palpable mass, and bloody occult stool are among some other symptomatic presentations of this carcinoma. Since the small intestinal products are liquid, obstruction is less likely, and stenosis-related symptoms are seldom detected in early SBA stages, leading to the late diagnosis at an advanced stage [4,9-11]. Surgical interventions such as resection are successful in 40%-60% of affected individuals [12-14]. The effectiveness of adjunctive chemotherapy in patients undergoing surgical resection is still debated, while progressed SBA has a five-year survival rate of 42% [15]. This paper reports a symptomatically and diagnostically challenging SBA case with iron deficiency anemia, that was successively diagnosed and treated.

## Case presentation

A 66-year-old male patient presented with a two-year history of physical weakness, anorexia, weight loss, and pallor with no complaints or history of hematemesis, melena, jaundice, or bleeding disorders. His systemic history was insignificant. On the patient's general physical examination, he was grossly pale, asthenic, and wasted. There was no discrete mass or palpable visceromegaly except mild fullness felt in the upper abdomen. Examination of hernial orifices and the inguinoscrotal region was unremarkable. The rest of the systemic examination was insignificant. Laboratory investigation revealed iron deficiency anemia along with features of chronic blood loss. Upper and lower GI endoscopies were normal. A stool microscopic examination showed occult blood. Tumor marker CA-125 was in the normal value of 7.270U/mL with a raised CRP of 8.800mg/L. A Tc-99m RBC scintigraphy conclusion was a probability of active GI bleed in the caecum and proximal ascending colon.

An abdominal CT scan with contrast showed normally appearing small and large bowels with no wall thickening, no mass lesion, and no enlarged mesenteric nodes. An esophagogastroduodenoscopy detected antral erosions with decreased Kerckring folds. A colonoscopy was also performed and showed normal mucosae, vascular patterns, and distensibility up to the terminal ileum. Biopsies showed a mild to moderate degree of chronic non-specific inflammation in the duodenum and caecum with chronic superficial gastritis and lymphoid aggregates in the gastric antrum. A small bowel follow-through report suggested thickening of the terminal ileum, caecum, and ascending colon with no dilated bowel loops or strictures. The test of anti-tissue transglutaminase (IgA, IgG) serum levels ruled out celiac disease.

During the investigation of anemia, he underwent bone marrow aspiration and trephine biopsy, which indicated hypocellularity, micronormoblastic dyserythropoiesis, and an increased number of hypolobulated megakaryocytes and micromegakaryocytes. There were no abnormal cells and iron absence in developing erythroblasts suggesting iron deficiency anemia. He was transfused and discharged on iron supplements. For two years, he was transfused 35 times and given iron supplements without improvement.

**Figure 1:**
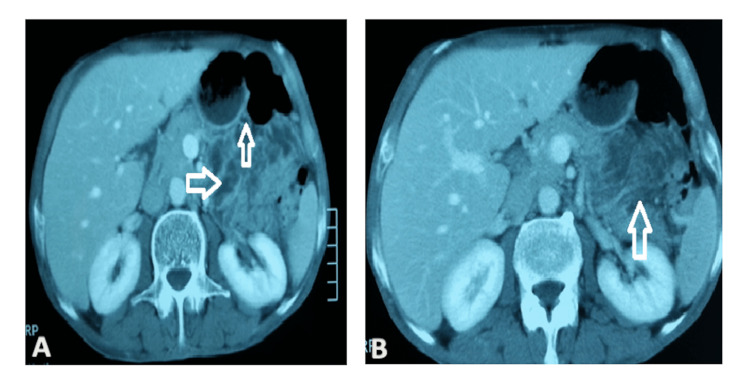
(A, B) Biphasic CT (axial view). The arrows in both panels are showing air in multiple small bowel loops indicating obstruction.

Another colonoscopy was performed, which stated that the colonic mucosa was normal, and grade 1 internal hemorrhoids were seen. A biphasic CT scan was performed through the abdomen and pelvis on a GI bleeding protocol and showed features of aneurysmal dilatation of the jejunal mid-segment with a significantly thick wall and obvious adjacent hypervascularity concerning possible lymphoma/MALToma.Figures 1A, 1B also show air in multiple small bowel loops indicating the possibility of obstruction.

Given the chronic blood loss, iron deficiency anemia, weight loss, and a possibility of a tumor on the biphasic CT scan report, a multidisciplinary approach from oncological, hematological, surgical, and radiological specialties was taken, and an exploratory laparotomy was planned.

Prior to the surgery, the patient's laboratory workup was done. A complete blood examination showed a high ESR of 62mm/lst hour, microcytic hypochromic anemia (hemoglobin: 8.7g/dL, HCT: 27.5%, MCV: 69.3% fL), increased platelet count (750×10^3^/µL) and a normal differential leukocyte count. His coagulation profile, liver function tests, renal profile, urine culture, and serum electrolytes were all within the normal ranges. A few days later, after optimization of the anemia, the new complete blood examination showed improved hemoglobin of 9.9g/dL. Then, the patient was operated on.

**Figure 2:**
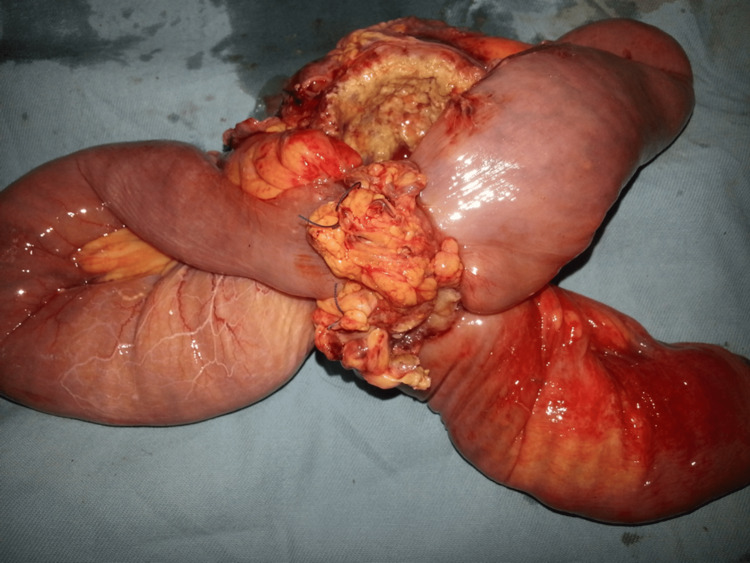
A resected jejunum showing the tumor and the fused loops of bowel.

The patient underwent exploratory laparotomy, and segmental resection of the jejunum [A1] was done. The resected jejunum measured 41cm long and had fused loops, as shown in Figure 2, forming a fistula with interconnected lumen due to the tumor, which measured 7.8cm×4.4cm×4.0cm. The tumor also had perforated serosa and invaded the adjacent bowel loop. The surgical specimen was sent for a histopathology test.

Histopathology analysis showed moderately differentiated adenocarcinoma. Surgical resection margins were free of tumor, the lymphovascular invasion was seen, and two out of 11 lymph nodes were positive for metastatic carcinoma. The postoperative period was uneventful, and the patient was discharged on the 10th postoperative day. During follow-up appointments, he remained well and received the FOLFOX regimen and adjuvant chemotherapy.

## Discussion

We presented a SBA case diagnosed after exploratory laparotomy and jejunectomy. All the preoperative diagnoses led to a nonspecific diagnosis rather than a misleading one considering there was non-specific chronic inflammation in the duodenum, caecum, and ascending colon without dilated bowel loops or strictures on repeated diagnostic tests. The biphasic CT scan was performed to rule out lymphoma and MALToma. Usually, small bowel lymphoma shows bowel wall thickening of 1-7 cm [16], and the MALToma also shows thickening of 2-4 cm on CT [17], whereas our patient showed no wall thickening. For SBA, the sensitivity of bowel barium transit is approximately 50%, and the CT scans have a precision of 47% [18].

The GI bleeding in the caecum, evident on Tc-99m RBC scintigraphy, unexplained weight loss, negative serum anti-tissue transglutaminase, and hypocellularity on bone marrow biopsy, pointed toward the possibility of a tumor in our case. The unexpected symptom was iron deficiency anemia, a rare occurrence and presenting signs in SBA. However, this is similar to a case report by Poddar et al., who reported an SBA case with refractory iron deficiency anemia, which was diagnosed after a year of investigations [19].

The risk factors for SBA are alcohol, cigarette smoking, family history, and environmental exposure. People like farm laborers, dry cleaners, welders, housekeepers, and caretakers are more likely to develop SBA due to their occupational exposure [20]. Dietary risk factors include red meat, processed meat, and refined carbohydrates. However, there is ambiguous evidence for these dietary risk factors. The SBA also tends to be due to a genetic predisposition involving a germline mutation in DNA mismatch repair (MMR) genes and also an association with Celiac disease, Crohn’s disease, and adenomas. However, in our case, Celiac disease was ruled out. Crohn's disease associated with SBA usually involves the ileum. However, in our case, the SBA involved the jejunum and duodenum.

According to Hong et al., patients receiving palliative chemotherapy showed a more significant survival rate compared to the ones who did not opt for it (p = 0.025) [11]. FOLFOX is considered one of the alternatives as effective platinum-based chemotherapy for SBA, and it is what our patient was taking during the follow-up visits showing positive progress and recovery.

## Conclusions

For an early and accurate diagnosis of SBA, efficient and timely clinical judgment by the physicians/surgeons is of prime importance leading to an improved chance of patient survival. Due to its various symptoms related to other diseases, SBA is a diagnostic challenge, especially when presented with rare symptoms like iron deficiency anemia. Delayed recognition at an advanced stage, where resection of a tumor is not beneficial, mostly results in a poor prognosis of the disease. As in our case, palliative chemotherapy shows a satisfactory improvement in the course of the disease.
